# Discovery of a Hepatoprotective Trinor-Sesterterpenoid from the Marine Fungus *Talaromyces* sp. Against Hepatic Ischemia-Reperfusion Injury

**DOI:** 10.3390/md23080329

**Published:** 2025-08-16

**Authors:** Wenxun Lan, Jian Cai, Liyan Yan, Xinyi Wu, Lisha Zhang, Chunmei Chen, Zhongqiu Liu, Xuefeng Zhou, Lan Tang

**Affiliations:** 1 Guangdong Provincial Key Laboratory of New Drug Screening, Guangdong-Hong Kong-Macao Joint Laboratory for New Drug Screening, School of Pharmaceutical Sciences, Southern Medical University, Guangzhou 510515, China; 13535996639@163.com (W.L.); liyanyan20231225@163.com (L.Y.); 19865809105@163.com (X.W.); 18897717406@163.com (L.Z.); 2 Guangdong Key Laboratory of Marine Materia Medica, State Key Laboratory of Tropical Oceanography, South China Sea Institute of Oceanology, Chinese Academy of Sciences, Guangzhou 510301, China; caijian@scsio.ac.cn (J.C.); chenchunmei18@mails.ucas.ac.cn (C.C.); 3 University of Chinese Academy of Sciences, Beijing 100049, China; 4 Joint Laboratory for Translational Cancer Research of Chinese Medicine of the Ministry of Education of the People’s Republic of China, International Institute for Translational Chinese Medicine, Guangzhou University of Chinese Medicine, Guangzhou 510006, China; liuzq@gzucm.edu.cn

**Keywords:** mangrove-derived fungus, talaromyces, hepatic ischemia-reperfusion injury

## Abstract

A new trinor-sesterterpenoid penitalarin D (**1**), with a 3,6-dioxabicyclo[3.1.0]hexane moiety, as well as two known compounds, penitalarin C (**2**) and nafuredin A (**3**), were obtained from the mangrove sediment-derived *Talaromyces* sp. SCSIO 41412. Their structures were determined by detailed NMR, MS spectroscopic analyses, and ECD calculations. Penitalarin D (**1**) and nafuredin A (**3**) showed toxicity or no toxicity against HepG2 cells at a concentration of 200 μM. The transcriptome sequencing and bioinformatics analysis revealed that **3** could be effective by regulating ferroptosis pathways in HepG2 cells, which was subsequently validated by RT-qPCR, demonstrating significant upregulation of ferroptosis-related genes. Pre-treatment with **3** could mitigate hypoxia-reoxygenation-induced damage in the oxygen glucose deprivation/reperfusion (OGD/R) cell model. Given the structural similarity of compounds **1**, **2**, and **3**, we also screened compounds **1** and **2** in an AML12 OGD/R model. As no significant activity was observed, compound **3** was selected for subsequent in vivo studies. Subsequently, in vivo experiments demonstrated that **3** could significantly decrease pro-inflammatory cytokines and display the hepatoprotective effects against hepatic ischemia-reperfusion injury (HIRI). These findings identified nafuredin A (**3**) as a promising hepatoprotective agent for new drug development.

## 1. Introduction

Hepatic ischemia-reperfusion injury (HIRI) represents a major clinical complication in liver transplantation, resection, and other surgical procedures, frequently leading to graft failure, tissue damage, and hepatic dysfunction [1]. This biphasic pathological process initiates with hypoxia-induced cellular injury during ischemia, which paradoxically exacerbates upon oxygen restoration during reperfusion. The reperfusion phase triggers a cascade of destructive events, including mitochondrial dysfunction, excessive reactive oxygen species (ROS) production, cytochrome C release, and ultimately hepatocyte death [2]. Currently, no effective pharmacological interventions exist for HIRI prevention or treatment [3], highlighting the urgent need to elucidate the molecular mechanisms underlying hepatic I/R injury to develop targeted therapies.

Ferroptosis, a novel form of regulated cell death characterized by iron overload and lipid peroxidation, has been implicated in various pathological conditions, including periventricular leukomalacia, cardiovascular diseases, liver disorders, renal diseases, cancer, and Huntington’s disease [4]. Emerging evidence particularly highlights its critical role in exacerbating ischemia-reperfusion (I/R) injury across multiple organ systems. Notably, pharmacological inhibition of ferroptosis using agents such as ferrostatin-1 has demonstrated significant protective effects in both renal and cardiac I/R models, effectively attenuating tissue damage and lipid peroxidation upon reperfusion [5]. These findings strongly suggest that targeting ferroptosis may offer a promising therapeutic strategy for hepatic ischemia-reperfusion injury (HIRI), with considerable potential for clinical translation in liver protection and regeneration.

Marine microorganisms, including those derived from the coastal mangrove ecosystems, represent a rich source of structurally diverse bioactive metabolites with significant pharmaceutical potential [6,7]. These microorganisms produce unique secondary metabolites that can modulate specific cellular pathways and biochemical processes, making them valuable candidates for drug discovery [8].

**Figure 1:**
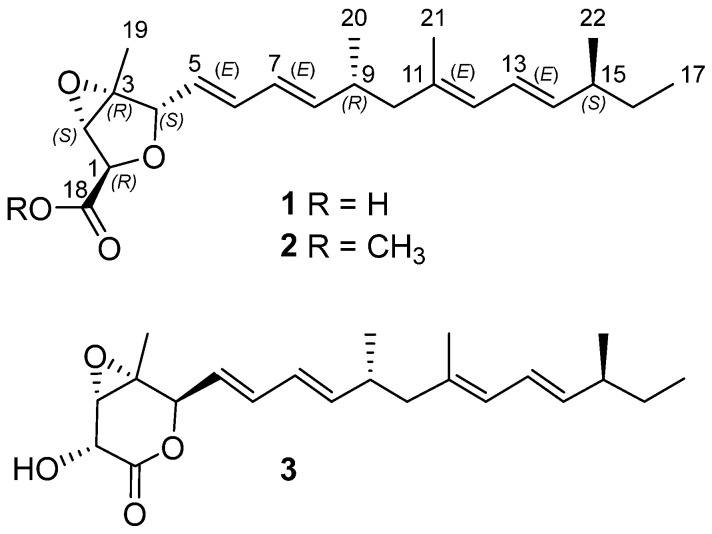
Structures of compounds **1**–**3**.

Herein, three trinor-sesterterpenoids (**1**–**3**, Figure 1) were isolated from the mangrove sediment-derived fungus *Talaromyces* sp. SCSIO 41412. This study described the isolation procedures, structural characterization, and pharmacological activity evaluation of the isolated compounds.

## 2. Results and Discussion

### 2.1. Structural Determination

**Figure 2:**
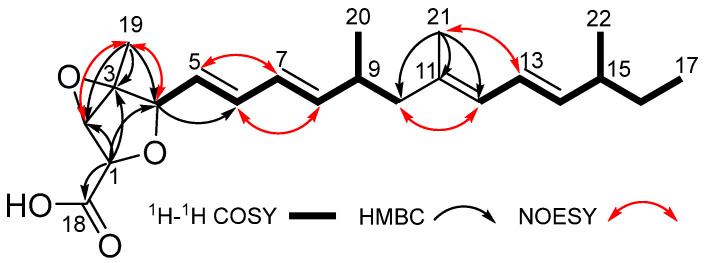
Key ^1^H-^1^H COSY, HMBC, and NOESY correlations of **1**.

Compound **1** was obtained as a yellow oil. Its molecular formula was determined as C_22_H_32_O_4_ by HRESIMS data, with an unsaturation degree of 7. The ^1^H NMR data (Table 1) showed five methyls [*δ*_H_ 0.81 (t, *J* = 7.4, H_3_-17), 1.32 (s, H_3_-19), 0.92 (d, *J* = 6.7 Hz, H_3_-20), 1.67 (s, H_3_-21), 0.95 (d, *J* = 6.7 Hz, H_3_-22)], two methylenes [*δ*_H_ 2.06/1.94 (H_2_-10), 1.28 (H_2_-16)], five *sp*^3^ methines with three of them being attached to oxygen atoms [*δ*_H_ 4.36 (overlapped, H-1), 3.92 (s, H-2), 4.37 (d, *J* = 8.1 Hz, H-4), 2.41 (m, H-9), 2.06 (overlapped, H-15)], and seven olefinic protons [5.65 (overlapped, H-5), 6.25 (dd, *J* = 15.3, 10.5 Hz, H-6), 6.02 (dd, *J* = 15.3, 10.4 Hz, H-7), 5.65 (overlapped, H-8), 5.75 (d, *J* = 10.8 Hz, H-12), 6.18 (dd, *J* = 15.1, 10.8 Hz, H-13), 5.43 (dd, *J* = 15.1, 7.8 Hz, H-14)]. In addition to the aforementioned 19 carbon signals connected by protons, the ^13^C NMR and HSQC data also showed three additional carbon signals, including one carbonyl carbon at *δ*_C_ 171.7 (C-18), one olefinic carbon at *δ*_C_ 134.1 (C-11), and one quaternary carbon at *δ*_C_ 65.3 (C-3). Analysis of the above HRESIMS and NMR spectra of **1** revealed similarity to penitalarin A (**2**) [9], except that **1** lacked one methoxy signal. Combined with the two spin systems [H-4/H-5/H-6/H-7/H-8/H-9/H_2_-10 (H_3_-20) and H-12/H-13/H-14/H-15/H_2_-16 (H_3_-22)/H_3_-17] observed in the ^1^H-^1^H COSY spectrum (Figure 2), as well as the HMBC correlations of H_3_-21/C-10, C-11, C-12, confirmed the presence of C4–C17 long chain. The HMBC correlation of H-19/C-2, C-3, C-4, and H-1/C-2, C-3, C-4, C-18 confirmed the presence of a hydrogenated furan moiety with a carboxyl group. Based on the above speculation, the planar structure of **1** was determined as shown in Figure 1.

**Figure 3:**
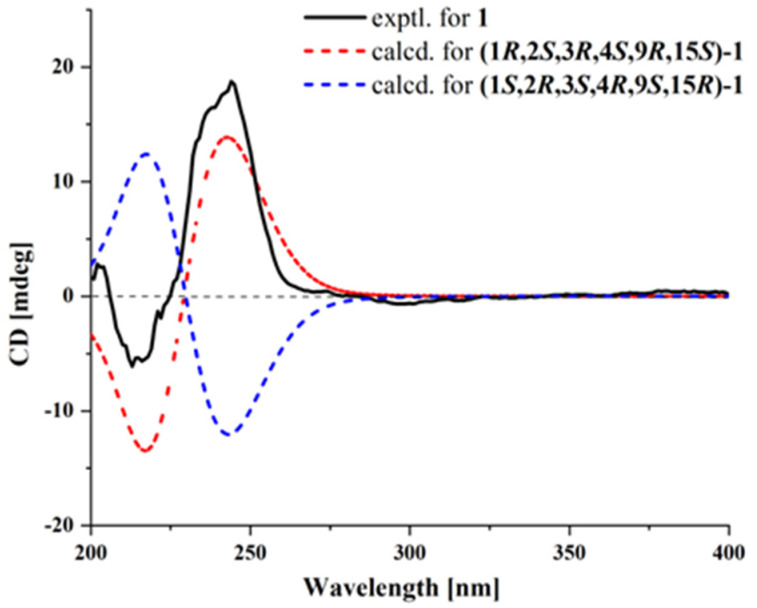
Experimental and calculated ECD spectra of **1**.

The relative configuration of **1** was established by NOESY correlations and coupling constants. The NOESY correlations of H-2, H-4/H_3_-19 indicated that they were oriented in the same direction. The absence of coupling between H-1 and H-2 suggested their *trans* relative configuration. According to the NMR data and biosynthetic pathway, the configurations of C-9 and C-15 in **1** were presumed to be the same as those of the known **2**. The coupling constants of *J*_H-5/H-6_ (15.3 Hz), *J*_H-7/H-8_ (15.3 Hz), and *J*_H-13/H-14_ (15.1 Hz) indicated that the configurations of double bonds Δ^5^, Δ^7^, Δ^13^ were *E*. Given that the chemical shift at C-21 was 16.3 ppm, the configuration of Δ^11^ was inferred to be *E* based on the *γ*-gauche effect. Furthermore, the NOESY correlations between H-5/H-7, H-6/H-8, H_2_-10/H-12, and H-13/H_3_-21 also confirmed that the conformations of the conjugated double bonds were *s-trans*. Therefore, the relative configurations were presumed to be *rel*-(1*R*,2*S*,3*R*,4*S*,9*R*,15*S*). Combined with ECD calculation (Figure 3), the absolute configuration of **1** was determined as 1*R*,2*S*,3*R*,4*S*,9*R*,15*S*, and it was named penitalarin D (**1**).

Additionally, the other two known penitalarin A (**2**) and nafuredin A (**3**) were studied by comparing their one-dimensional NMR data with those reported in the literature [9].

### 2.2. Bioactivity Assay

**Figure 4:**
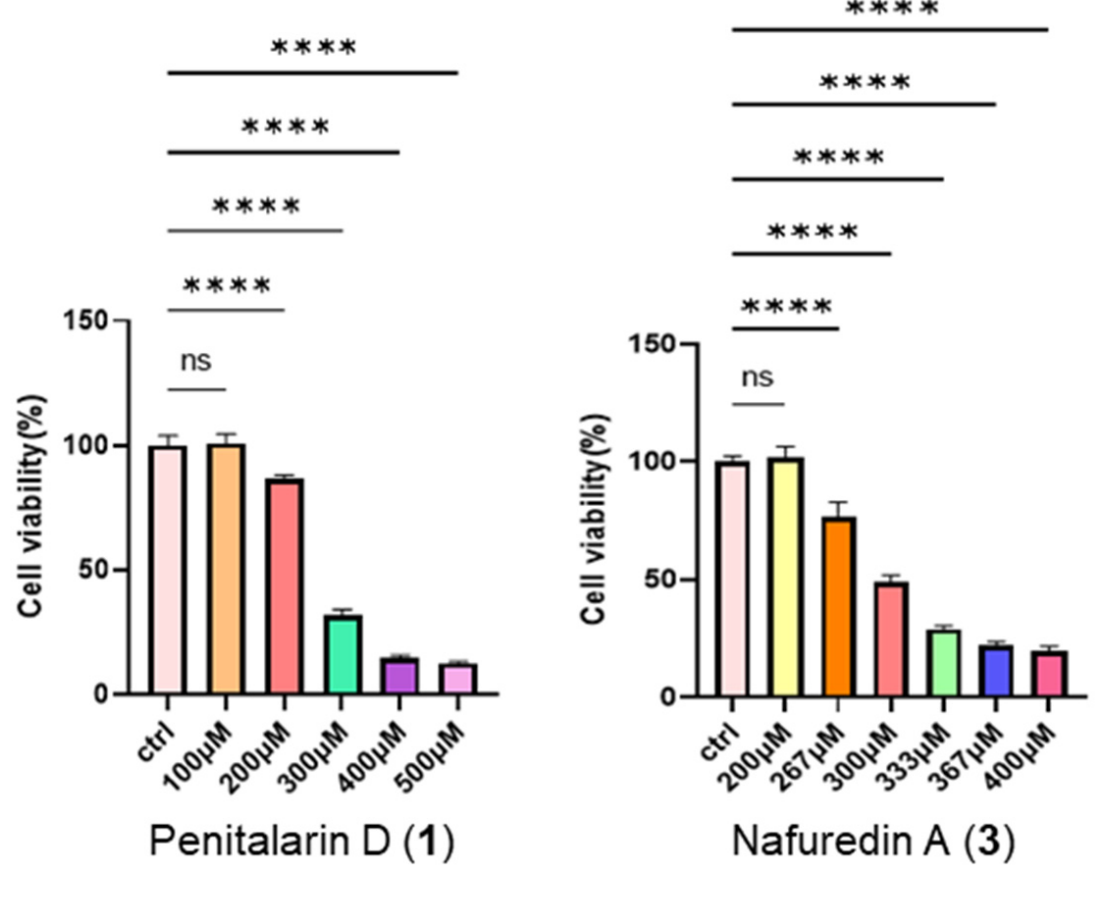
Weak cytotoxic effects of compounds **1** and **3** on HepG2 cells (*n* = 5). **** *p* < 0.0001, ns: no significance between indicated groups. (One Way Anova).

This study investigated the pharmacological activities of the trinor-sesterterpenoids (**1**–**3**). Initial cytotoxicity assessment using CCK-8 assays revealed no or minimal toxicity in HepG2 cells at 200 μM (Figure 4), with IC_50_ values of 270.7 μM for **1** and 303.6 μM for **3** (Figure S10).

**Figure 5:**
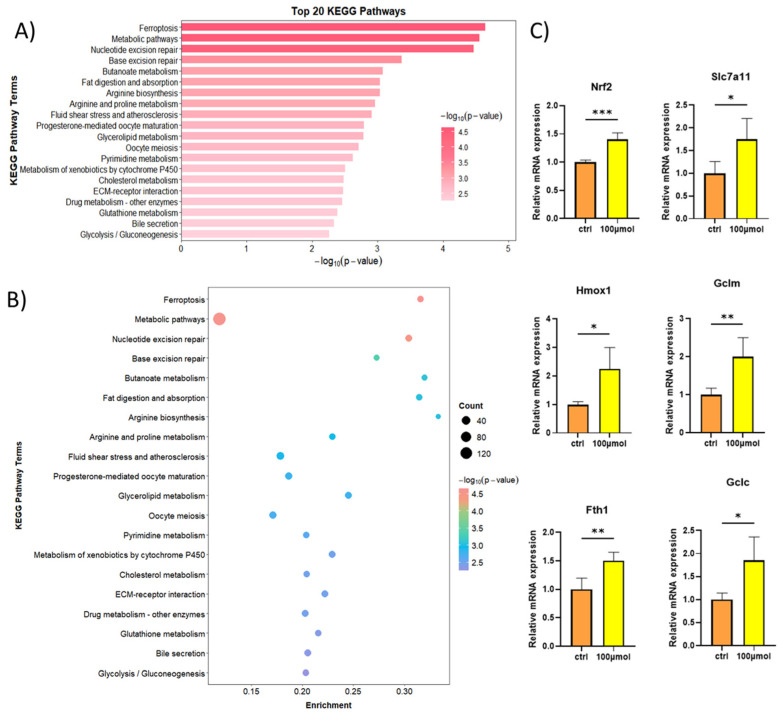
(**A**,**B**) RNA-seq analysis of HepG2 cells after **3** treatments. (**C**) RT-qPCR analysis of *SLC7A11*, *HMOX1*, *NRF2*, *FTH1*, *GCLC*, and *GCLM* mRNA levels in HepG2 cells treated with **3** (200 μM) (*n* = 4). * *p* < 0.05, ** *p* < 0.01, *** *p* < 0.001.

Due to the limited availability of **1**, we selected **3** (200 μM, 24 h treatment) for transcriptomic analysis in HepG2 cells. KEGG pathway enrichment analysis identified ferroptosis as a top-ranked pathway of interest (Figure 5A,B), prompting further mechanistic investigation. Subsequent validation in HepG2 cells treated with compound **3** (100 μM, 24 h) revealed robust upregulation of core ferroptosis regulatory genes—NRF2, SLC7A11, FTH1, HMOX1, GCLC, and GCLM—via RT-qPCR analysis (Figure 5C). Ferrostatin-1 was a potent and selective ferroptosis inhibitor that did not directly affect SLC7A11 activity or expression. Conversely, the coordinated transcriptional changes induced by compound **3** demonstrated its effective activation of the NRF2/SLC7A11 axis, establishing its role in suppressing lipid peroxidation and late-stage cell death. Kelch-like ECH-associated protein 1 (KEAP1)/nuclear factor erythroid 2-related factor 2 (NRF2), a well-characterized protein complex, regulates the expression of numerous antioxidant genes that maintain cellular oxidative stress homeostasis. Under physiological conditions, KEAP1 binds to cytoplasmic NRF2, promoting its ubiquitination and subsequent degradation. We hypothesize that **3** activates NRF2 by competitively binding to KEAP1, thereby disrupting the KEAP1-NRF2 interaction, enhancing NRF2 nuclear translocation, and upregulating the transcription of downstream antioxidant genes. To further elucidate the binding mode of compound **3** with the KEAP1-NRF2 complex, an in silico molecular docking analysis was performed using a KEAP1-NRF2 homology model (PDB code: 5WHL). The docking results revealed that compound **3** is well accommodated within the binding pocket of KEAP1-NRF2, exhibiting a binding score of −5.352 kcal/mol. The 2D binding model demonstrates that the hydroxyl group of **3** forms a hydrogen bond with the active site residue Val608. These findings suggest that compound **3** may competitively displace the KEAP1-NRF2 complex, thereby potentially modulating the expression of downstream genes (Figure S11 and Table S2) [10,11].

**Figure 6:**
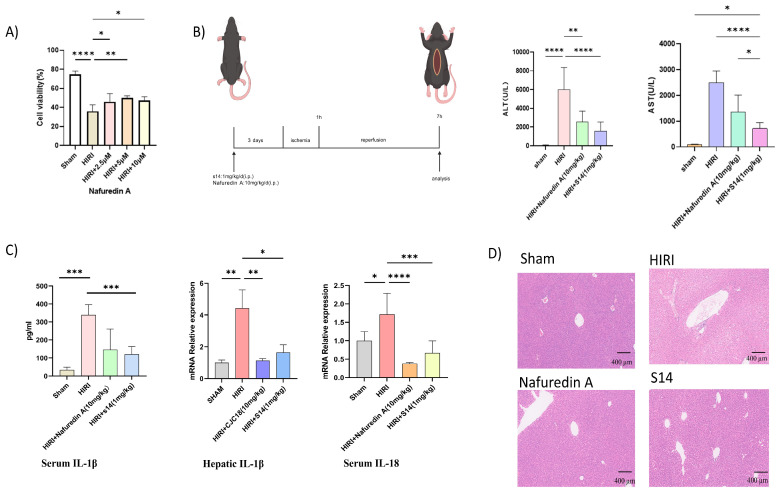
Nafuredin A (**3**) alleviates OGD/R-induced AML12 cell injury and mitigates HIRI in mice. (**A**) Cell viability assay (*n*  =  5). (**B**) Flow chart of animal experiments. (**C**) Serum levels of AST and ALT in mice (*n*  =  6). (**D**) Serum IL-1β concentrations (*n* = 6) and hepatic IL-1β/IL-18 mRNA expression levels (*n* = 5) were measured in murine models. Representative images of H&E-stained mouse liver tissue (scale bar = 400 µm). * *p* < 0.05, ** *p* < 0.01, *** *p* < 0.001, **** *p* < 0.0001.

Given the established association of the ferroptosis pathway with various organ pathologies, including Huntington’s disease, cardiac disorders, and hepatic/renal diseases [12,13,14], we specifically examined its role in hepatic ischemia-reperfusion injury (HIRI). To evaluate the hepatoprotective effects of nafuredin A (**3**) against metabolic stress, AML12 cells were pretreated with compound **3** (2.5–10 μM) for 24 h prior to establishing an oxygen-glucose deprivation/reperfusion (OGD/R) injury model in vitro. Nafuredin A (**3**) significantly attenuated OGD/R-induced cytotoxicity in a concentration-dependent manner, as evidenced by the restoration of cellular viability (Figure 6A). Considering that compounds **1** and **2** are analogues, we also performed the in vitro OGD/R injury model with AML12 cells. However, these two compounds showed no detectable activity. Therefore, we selected compound **3** for subsequent in vivo experiments. In a murine hepatic ischemia-reperfusion injury (HIRI) model (Figure 6B), pretreatment with nafuredin A (**3**) (10 mg/kg/day, i.p.) administered for **3** consecutive days prior to ischemia induction significantly attenuated hepatocellular damage compared to untreated HIRI controls, as evidenced by ≥45% reductions in serum alanine aminotransferase (ALT) and aspartate aminotransferase (AST) levels (Figure 6C). It was revealed that nafuredin A (**3**) could significantly decrease pro-inflammatory cytokines, as measured by the measurement of serum IL-1β concentrations and hepatic IL-1β/IL-18 mRNA expression levels (Figure 6D). Compound **3** treatment substantially attenuated HIRI-induced hepatic histoarchitectural disruption, as demonstrated by representative H&E-stained sections. Key morphological improvements included markedly diminished nuclear pyknosis, indicating reduced cellular degeneration; restoration of sinusoidal endothelial integrity with resolution of pathological congestion; and reorganization of hepatic cords into their characteristic radiating architecture. These collective changes—reflecting mitigation of both cytological damage and tissue-level disorganization—corroborated the observed functional recovery in serum biomarkers, confirming compound **3**’s structural preservation capacity under ischemic stress. Notably, the therapeutic efficacy of nafuredin A (**3**) was comparable to that of S14, another marine-derived small-molecule compound previously identified by our group as an effective treatment for acute kidney injury (AKI) [15], which served as the positive control in this study. These findings collectively demonstrate that nafuredin A (**3**) exerts hepatoprotective effects against HIRI, potentially through modulation of the NRF2/ferroptosis pathway.

## 3. Materials and Methods

### 3.1. General Experimental Procedures

Optical rotations were measured on an Anton Paar MPC 500 polarimeter. UV spectra were recorded using a Shimadzu UV-2600 PC spectrometer (Beijing, China), while IR spectra were obtained with an IR Affinity-1 spectrometer. NMR analyses were conducted on a Bruker Avance spectrometer, operating at 500 MHz for ^1^H NMR and 125 MHz for ^13^C NMR. HRESIMS data were acquired via a Bruker maXis Q-TOF mass spectrometer (Fällanden, Switzerland). HPLC experiments were performed on a Hitachi Primaide system (Tokyo, Japan) equipped with a DAD detector, utilizing an ODS column (YMC-pack ODS-A, 10 × 250 mm, 5 μm).

### 3.2. Fungal Material

A fungal strain designated as *Talaromyces* sp. SCSIO 41412 was isolated from a sediment sample collected at the Gaoqiao Mangrove in Zhanjiang City, Guangdong Province, China, in August 2021. Taxonomic identification of the fungus was performed by analyzing the internally transcribed spacer (ITS) region of ribosomal DNA (rDNA), with the ITS sequence deposited in GenBank under accession number PP001498. The strain is currently preserved on malt extract agar slants (15 g malt extract, 18 g agar, 10 g sea salt, 1 L water) at 4 °C.

### 3.3. Fermentation, Extraction, and Isolation

The fungal strain *Talaromyces* sp. SCSIO 41412 was cultivated in 200 mL of seed medium containing 15 g malt extract, 10 g sea salt, and 1 L of water. The culture was incubated on a rotary shaker at 180 rpm and 28 °C for 3 days. The resulting seed culture was used to inoculate a large-scale fermentation system consisting of forty-seven Erlenmeyer flasks, each containing a rice medium (200 g rice, 2% sea salt, and 230 mL water). The fermentation was conducted under static conditions at 26 °C for 28 days. Following fermentation, the entire culture was extracted with ethyl acetate (EtOAc). The EtOAc extract was subjected to silica gel column chromatography, eluted with gradient mixtures of CH_2_Cl_2_/petroleum ether (0–100%) and CH_3_OH/CH_2_Cl_2_ (0–100%), yielding ten fractions (Frs. 1–10).

Fr.3 was subjected to ODS silica gel chromatography and eluted with CH_3_OH/H_2_O (5%–100%) to yield 10 subfractions. Fr.3–4 was further separated by semi-preparative HPLC (83% CH_3_OH/H_2_O, 3 mL/min) to afford compounds **1** (15.0 mg, *t*_R_ = 20.8 min) and **3** (105.2 mg, *t*_R_ = 16.0 min). Fr.4 was subjected to ODS silica gel chromatography and eluted with CH_3_OH/H_2_O (5%–100%) to yield 14 subfractions. Fr.4–14 was separated by semi-preparative HPLC (90% CH_3_OH/H_2_O, 2.5 mL/min) to yield **2** (5.5 mg, *t*_R_ = 16.9 min).

### 3.4. Spectroscopic Data of Compound ***1***

Penitalarin D (**1**): yellow oil; ${\left[\alpha \right]}_{D}^{25}$ +34.7 (*c* 0.1, CH_3_OH); ECD (0.3 mg/mL, CH_3_OH) *λ*_max_ (Δ*ε*) 213 (−2.23), 244 (+6.83); UV (CH_3_OH) *λ*_max_ (log *ε*) 200 (4.03), 210 (3.90), 230 (4.08) nm; IR *ν*_max_ 3429. 2960, 2929, 2873, 1716, 1456, 1377, 1207, 1072, 974, 846 cm^−1^; ^1^H and ^13^C NMR, Table 1; HRESIMS *m*/*z* 359.2234 [M-H]^−^ (calcd for C_22_H_31_O_4_^−^, 359.2228).

### 3.5. ECD Computation Section

Compound **1** was subjected to conformational searching in Spartan’14 using the MMFF molecular force field. The stable conformers representing the top 5% of the potential isomers were then optimized in methanol solvent at the B3LYP/6-31G (d) level of theory using Gaussian 09. The optimized low-energy conformations were further analyzed using TDDFT with a polarizable continuum model at the B3LYP/6-311G (d, p) level [16]. The calculated electronic circular dichroism (ECD) spectra were generated from GaussView 6.0, with a half-bandwidth of 0.3 eV, wavelength-corrected by the calculated UV curve, and Boltzmann-weighted to obtain the final calculated ECD spectra.

### 3.6. Cell Culture and Treatment

HepG2 cells were purchased from the Shanghai Cell Bank, Chinese Academy of Sciences, and cultured in Dulbecco’s modified Eagle’s medium (DMEM, Gibco, New York, NY, USA) supplemented with 10% fetal bovine serum (FBS, ExCell Bio, Suzhou, China) at 37 °C under 5% CO_2_. The mouse hepatocyte AML12 cell line (Cell Bank of Chinese Academy of Sciences, Shanghai) was cultured under identical conditions. For oxygen-glucose deprivation/reperfusion (OGD/R) modelling, AML12 cells were subjected to oxygen-glucose deprivation in an anaerobic chamber (95% *n*_2_/5% CO_2_) for 4 h using glucose-free medium, followed by 6 h reperfusion with complete medium under normoxic conditions. For drug treatments, cells were pre-incubated with nafuredin A (**3**) (2.5, 5, or 10 μmol/L) in high-glucose medium for 24 h prior to experiments, with vehicle-treated cells serving as controls. Stock solutions (10 mM) of compounds **1** and **3** were prepared in dimethyl sulfoxide (DMSO, Tianjin Fuyu Fine Chemical Co., Ltd., Tianjin, China) and stored at −20 °C. Working concentrations were generated by diluting stocks in cell culture medium immediately before experimentation. Control groups received equivalent concentrations of DMSO (0.1% *v*/*v*) in culture medium.

### 3.7. Cytotoxic Bioassay

Cell viability was assessed using the CCK-8 assay (Dojindo, Kumamoto, Japan). Cells were plated at 3000 cells/well in 96-well plates and exposed to varying concentrations of compounds or solvent control. After 24 h incubation, CCK-8 reagent was added, and absorbance was measured at 450 nm using an Envision 2104 multilabel reader (PerkinElmer, Waltham, MA, USA). Dose–response curves were generated to determine IC_50_ values using Prism 9.5 software (GraphPad, San Diego, CA, USA).

### 3.8. RNA Sequencing Analysis

HepG2 cells were treated with nafuredin A (**3**) (200 μM, 24 h) or control. Total RNA was extracted using TRIzol reagent (Thermo Fisher (Waltham, MA, USA), 15596018), and quality was assessed (concentration > 50 ng/μL, RIN > 7.0, total RNA > 1 μg). Poly(A)-enriched mRNA was isolated, fragmented (94 °C, 5–7 min), and reverse-transcribed. Strand-specific libraries (300 ± 50 bp) were prepared and sequenced (PE150) on an Illumina NovaSeq 6000 platform (LC-Bio, Hangzhou, China).

### 3.9. RT-qPCR

Total RNA was extracted using RNAprep Kit (FOREGENE (Chengdu, China), RE-03014) and reverse transcribed using RT Master Mix (Takara (San Jose, CA, USA), RR037A). RT-qPCR was performed using SYBR Green Master Mix (Promega (Madison, WI, USA), A6002) with GAPDH normalization. Primers are listed in Table S1.

### 3.10. ELISA

Serum IL-1β concentrations were determined using a Murine IL-1β ELISA Kit (Abclonal (Woburn, MA, USA), Cat# RK04599) according to the manufacturer’s protocol: microplates underwent three washes with wash buffer (300 μL/well) prior to sequential addition of 50 μL biotinylated antibody working solution and 50 μL serum samples/standards; after 1 h incubation at 37 °C and three additional washes, 100 μL streptavidin-HRP working solution was added per well for 30 min at 37 °C; subsequent washes were followed by 100 μL substrate solution incubation (15–20 min, 37 °C, light-protected), terminated with 50 μL stop solution, with OD450 nm measured within 5 min (reference wavelength: 570 nm).

### 3.11. Animal Experiments

Male C57BL/6 mice (6–8 weeks old, 20–23 g) were procured from SPF (Beijing) Biotechnology Co., Ltd. After a 3-day acclimatization period in specific pathogen-free (SPF) facilities maintained at 25 ± 2 °C with 12 h/12 h light/dark cycles, mice were randomly allocated into four experimental cohorts (*n* = 6/group). All animals had ad libitum access to autoclaved chow and sterile water throughout the study protocol: Control, HIRI, HIRI+nafuredin A (**3**) (10 mg/kg), and HIRI+s14 (1 mg/kg). The HIRI model was established in C57BL/6 mice anesthetized with tribromoethanol (20 mL/kg, i.p.). Microvascular clamps were applied to the hepatic portal vein and hepatic artery to induce ischemia in ~70% of hepatic parenchyma (left/median lobes) for 1 h, followed by clamp removal to initiate 6 h reperfusion. Under terminal anesthesia (tribromoethanol, 20 mL/kg i.p.), serum was collected via cardiac puncture (centrifuged at 3000× *g*/10 min/4 °C) and stored at −80 °C, while liver tissues were harvested for either fixation in 4% PFA or snap-freezing in liquid nitrogen. Treatments were administered via intraperitoneal injection for 3 consecutive days prior to HIRI induction.

### 3.12. Serum Biochemical Analysis

Serum AST and ALT levels were measured using commercial kits (Jiancheng Bioengineering Institute, Nanjing, China).

### 3.13. Histopathological Examination

Hepatic tissue samples were fixed in 4% paraformaldehyde for 24 h at 4 °C, dehydrated through a graded ethanol series (70%, 80%, 95%, 100%), cleared in xylene, and embedded in paraffin. Serial sections (5 μm thickness) were mounted on glass slides, deparaffinized in xylene, and rehydrated through descending ethanol concentrations. Sections were stained with hematoxylin (5 min), differentiated in 1% acid alcohol (30 s), blued in 0.2% ammonia water (1 min), counterstained with eosin (3 min), then dehydrated through ascending ethanol and xylene prior to coverslipping with neutral balsam for microscopic evaluation.

### 3.14. Molecular Docking

The crystal structure of Keap-1 (PDB ID: 5WHL) was retrieved from the Protein Data Bank and prepared using the Protein Preparation Wizard workflow in Maestro 11.1 (Schrödinger Release 2017-1). Subsequently, the prepared ligand was flexibly docked into the receptor using the Induced Fit Docking module with default parameters.

### 3.15. Statistical Analysis

Data were analyzed using Prism 9.5 (GraphPad). Groups were compared by one-way ANOVA with significance set at *p* < 0.05 (* *p* < 0.05, ** *p* < 0.01, *** *p* < 0.001, **** *p* < 0.0001).

## 4. Conclusions

In this study, three natural trinor-sesterterpenoids (**1**–**3**), including a new compound named penitalarin D (**1**), were isolated from the mangrove sediment-derived fungus *Talaromyces* sp. SCSIO 41412. Through detailed structural characterization, their absolute configurations were elucidated. It was revealed that nafuredin A (**3**), with slight toxicity at 200 μM, can be effective by regulating ferroptosis pathways in HepG2 cells, through transcriptome sequencing and bioinformatics analysis. Subsequently, nafuredin A (**3**) was confirmed through its hepatoprotective effects against HIRI by in vitro and in vivo experiments, thereby demonstrating its potential for new drug development.

## Figures and Tables

**Table 1:** ^1^H (500 MHz) and ^13^C (125 MHz) NMR data of 1 in DMSO-*d*_6_.

Pos.	*δ*_C_ Type	*δ*_H_ (*J* in Hz)
1	76.7, CH	4.36, overlapped
2	63.7, CH	3.92, s
3	65.3, C	
4	82.6, CH	4.37, d (8.1)
5	128.6, CH	5.65, overlapped
6	133.7, CH	6.25, dd (15.3, 10.5)
7	127.3, CH	6.02, dd (15.3, 10.4)
8	141.3, CH	5.65, overlapped
9	34.2, CH	2.41, m
10	46.9, CH_2_	2.06, overlapped 1.94, m
11	134.1, C	
12	126.4, CH	5.75, d (10.8)
13	124.8, CH	6.18, dd (15.1, 10.8)
14	138.0, CH	5.43, dd (15.1, 7.8)
15	37.9, CH	2.06, overlapped
16	29.3, CH_2_	1.28, m
17	11.6, CH_3_	0.81, t (7.4)
18	171.7, C	
19	13.7, CH_3_	1.32, s
20	19.6, CH_3_	0.92, d (6.7)
21	16.3, CH_3_	1.67, s
22	20.0, CH_3_	0.95, d (6.7)

## Supplementary Materials

The following supporting information can be downloaded at: https://www.mdpi.com/article/10.3390/md23080329/s1, the physicochemical data of the known compounds **2** and **3**, Figures S1–S9: NMR, HRESIMS, UV, IR, and UV spectra of **1**, Figure S10: IC_50_ of **1** and **3** on HepG2 cells, Figure S11: the docking site of the Nafuredin A complex with Nrf2-keap1, Table S1: primers of RT-qPCR, Table S2: the docking site and docking scores of the **3** complex with Nrf2-keap1.
