# Supplementary material for: Discovery of a Hepatoprotective Trinor-Sesterterpenoid from the Marine Fungus Talaromyces sp. Against Hepatic Ischemia-Reperfusion Injury
Source: Mar Drugs. 2025 Aug 16;23(8):329. doi: 10.3390/md23080329 (PMC12387297; doi:10.3390/md23080329)
Supplement: Supplementary file 1 [file marinedrugs-23-00329-s001.zip › marinedrugs-3791431-supplementary.pdf]

Supporting Information

Discovery of a Hepatoprotective Trinor-Sesterterpenoid from the Marine Fungus *Talaromyces* sp. Against Hepatic Ischemia-Reperfusion Injury

Wenxun Lan, [1,†] Jian Cai, [2,3,†] Liyan Yan, [1] Xinyi Wu, [1] Lisha Zhang, [1] Chunmei Chen, [2,3] Zhongqiu Liu, [4] Xuefeng Zhou, [2,*] and Lan Tang [1,*]

[1] Guangdong Provincial Key Laboratory of New Drug Screening, Guangdong-Hong Kong-Macao Joint Laboratory for New Drug Screening, School of Pharmaceutical Sciences, Southern Medical University, Guangzhou 510515, China; 13535996639@163.com (W.L.); liyanyan20231225@163.com (L.Y.); 19865809105@163.com (X.W.); 18897717406@163.com (L.Z.)

[2] Guangdong Key Laboratory of Marine Materia Medica, State Key Laboratory of Tropical Oceanography, South China Sea Institute of Oceanology, Chinese Academy of Sciences, Guangzhou 510301, China; caijian@scsio.ac.cn (J.C.); chenchunmei18@mails.ucas.ac.cn (C.C.)

[3] University of Chinese Academy of Sciences, Beijing 100049, China

[4] Joint Laboratory for Translational Cancer Research of Chinese Medicine of the Ministry of Education of the People's Republic of China, International Institute for Translational Chinese Medicine, Guangzhou University of Chinese Medicine, Guangzhou 510006, China; liuzq@gzucm.edu.cn (Z.L.)

* Correspondence: xfzhou@scsio.ac.cn (X.Z.); tl405@smu.edu.cn (L.T.)

† These authors contributed equally to this work.

Contents

The physicochemical data of the known compounds **2** and **3**

Figures S1-S9: NMR, HRESIMS, UV, IR, and UV spectra of **1**

Figure S10. IC_{50} of penitalarin D (**1**) and nafuredin A (**3**) on HepG2 cells

Table S1. Primers of RT-qPCR

Figure S11. The docking site of the Nafuredin A complex with Nrf2-keap1

Table S2. The docking scores of the Nafuredin A complex with Nrf2-Keap1

The physicochemical data of the known compounds 2 and 3

Penitalarin A (**2**): yellow oil; ^{1}H NMR (500 MHz, DMSO-d_6) δ 6.26 (1H, dd, J = 15.2, 10.5 Hz, H-6), 6.17 (1H, dd, J = 15.1, 10.8 Hz, H-13), 6.03 (1H, dd, J = 15.3, 10.5 Hz, H-7), 5.75 (1H, d, J = 10.8 Hz, H-12), 5.66 (1H, dd, J = 15.3, 7.4 Hz, H-8), 5.60 (1H, dd, J = 15.2, 9.0 Hz, H-5), 5.43 (1H, dd, J = 15.1, 7.8 Hz, H-14), 4.50 (1H, s, H-1), 4.41 (1H, d, J = 9.0 Hz, H-4), 3.97 (1H, s, H-5), 3.69 (3H, s, H-19), 2.42 (1H, m, H-9), 2.06 (2H, dt, J = 13.5, 6.9 Hz, H-10), 1.95 (1H, m, H-15), 1.67 (3H, s, H-22), 1.33 (3H, s, H-20), 1.27 (2H, m, H-16), 0.93 (6H, m, H-23, 21), 0.81 (3H, t, J = 7.4 Hz, H-17); ^{13}C NMR (125 MHz, DMSO-d_6) δ 170.5 (C, C-18), 141.6 (CH, C-8), 138.1 (CH, C-14), 134.2 (C, C-11), 134.0 (CH, C-6), 127.9 (CH, C-5), 127.3 (CH, C-7), 126.5 (CH, C-12), 124.9 (CH, C-13), 82.6 (CH, C-4), 76.3 (CH, C-1), 65.0 (C, C-3), 63.2 (CH, C-2), 52.1 (CH_3, C-19), 46.9 (CH_2, C-10), 38.0 (CH, C-15), 34.3 (CH, C-9), 29.3 (CH_2, C-16), 20.1 (CH_3, C-23), 19.8 (CH_3, C-21), 16.3 (CH_3, C-22), 13.7 (CH_3, C-20), 11.7 (CH_3, C-17).

Nafuredin (**3**): yellow oil; ^{1}H NMR (500 MHz, Chloroform-d) δ 6.34 (1H, dd, J = 15.3, 10.6 Hz, H-7), 6.14 (1H, dd, J = 15.2, 10.9 Hz, H-14), 5.99 (1H, dd, J = 15.3, 10.3 Hz, H-8), 5.77 (1H, dd, J = 15.2, 7.1 Hz, H-9), 5.73 (1H, d, J = 11.3 Hz, H-13), 5.47 (1H, dd, J = 14.8, 7.8 Hz, H-6), 5.42 (1H, m, H-15), 4.89 (1H, d, J = 7.6 Hz, H-5), 4.57 (1H, s, H-2), 3.49 (2H, s, H-3), 2.40 (1H, m, H-10), 2.05 (2H, m, H-16), 1.93 (1H, dd, J = 13.6, 8.1 Hz, H-11), 1.67 (3H, s, 12-CH_3), 1.42 (3H, s, 4-CH_3), 1.28 (3H, q, J = 7.1 Hz, H-17), 0.95 (6H, t, J = 7.0 Hz, 10, 16-CH_3), 0.82 (3H, m, H-18); ^{13}C NMR (125 MHz, Chloroform-d) δ 170.7 (C, C-1), 145.1 (CH, C-9), 138.9 (CH, C-15), 138.0 (CH, C-7), 133.7 (C, C-12), 127.1 (CH, C-13), 126.2 (CH, C-8), 124.7 (CH, C-14), 122.2 (CH, C-6), 80.3 (CH, C-5), 68.1 (CH, C-2), 58.8 (CH, C-3), 58.4 (C, C-4), 47.3 (CH_2, C-11), 38.7 (CH, C-16), 34.9 (CH, C-10), 29.9 (CH_2, C-17), 20.2 (CH_3, C-16), 19.5 (CH_3, C-10), 17.8 (CH_3, C-4), 16.6 (CH_3, C-12), 11.8 (CH_3, C-18).

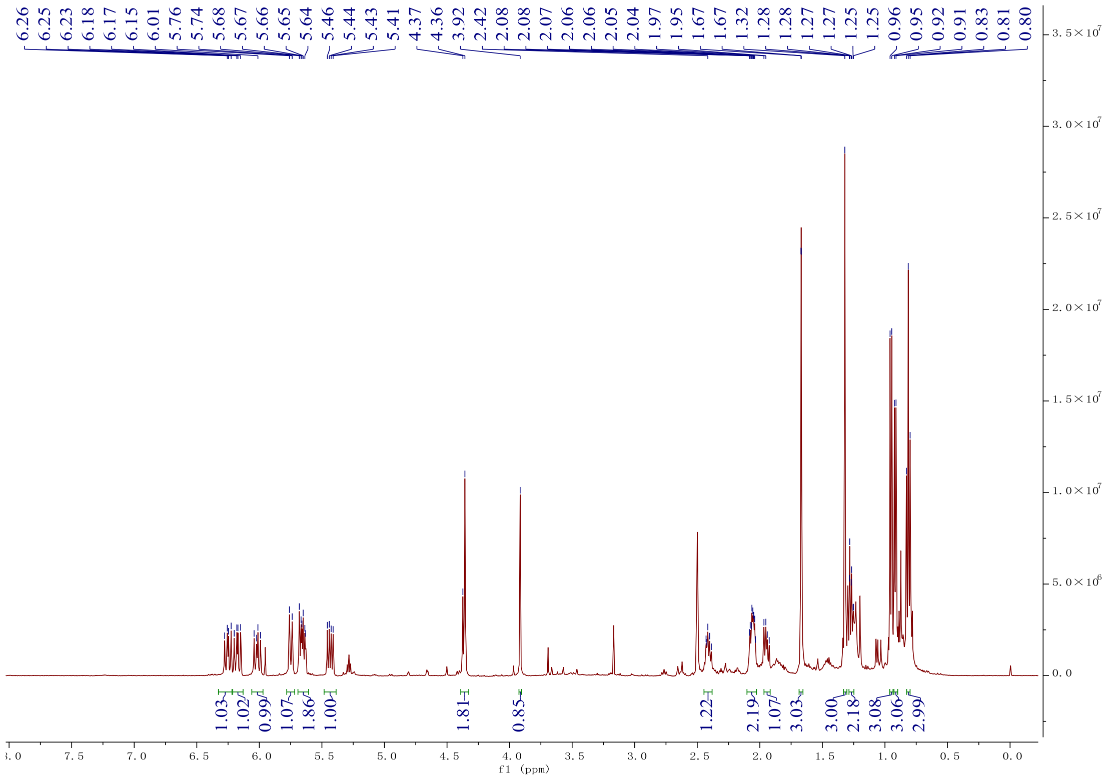

Figure S1. ^{1}H NMR (500 MHz) spectrum of **1** in DMSO-*d*6.

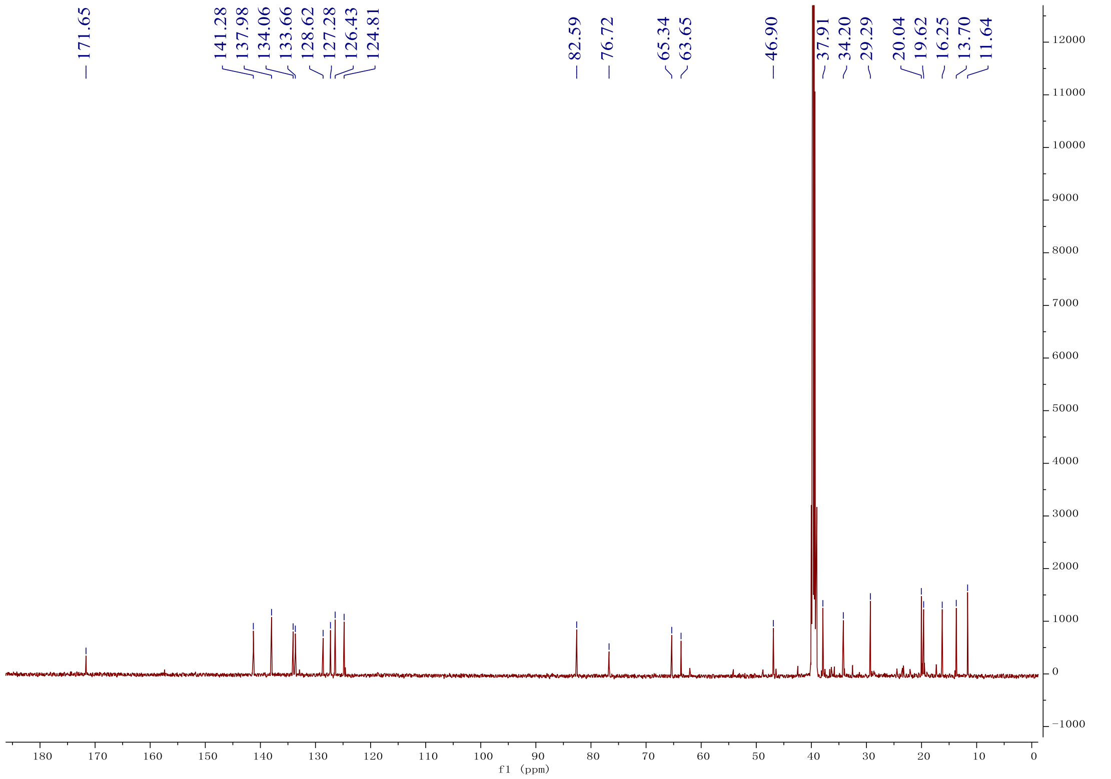

Figure S2. ^{13}C NMR (125 MHz) spectrum of **1** in DMSO-d_6.

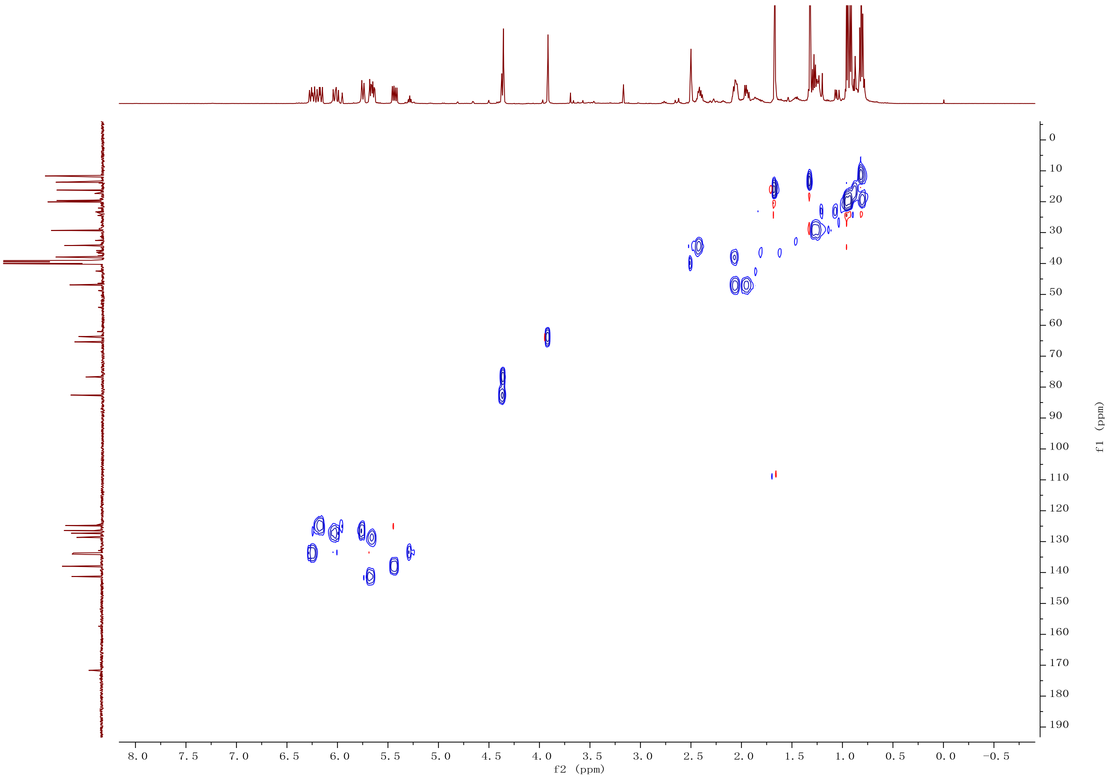

Figure S3. HSQC spectrum of **1** in DMSO-d_6.

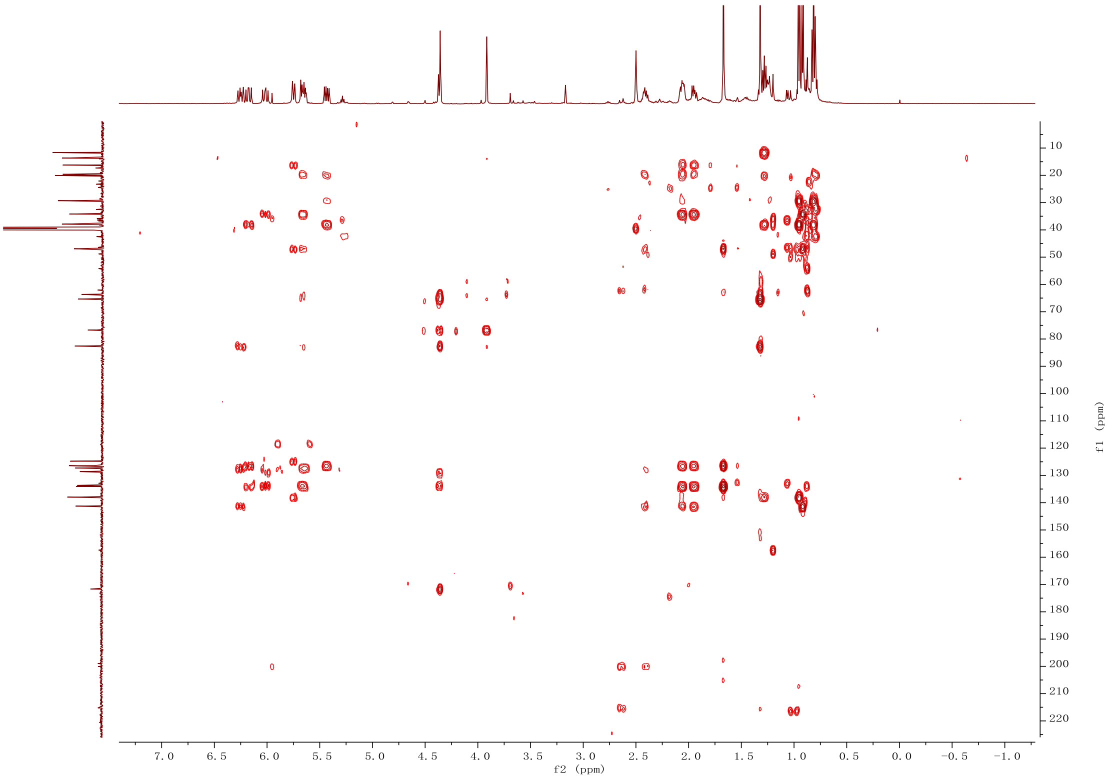

Figure S4. HMBC spectrum of **1** in DMSO-d_6.

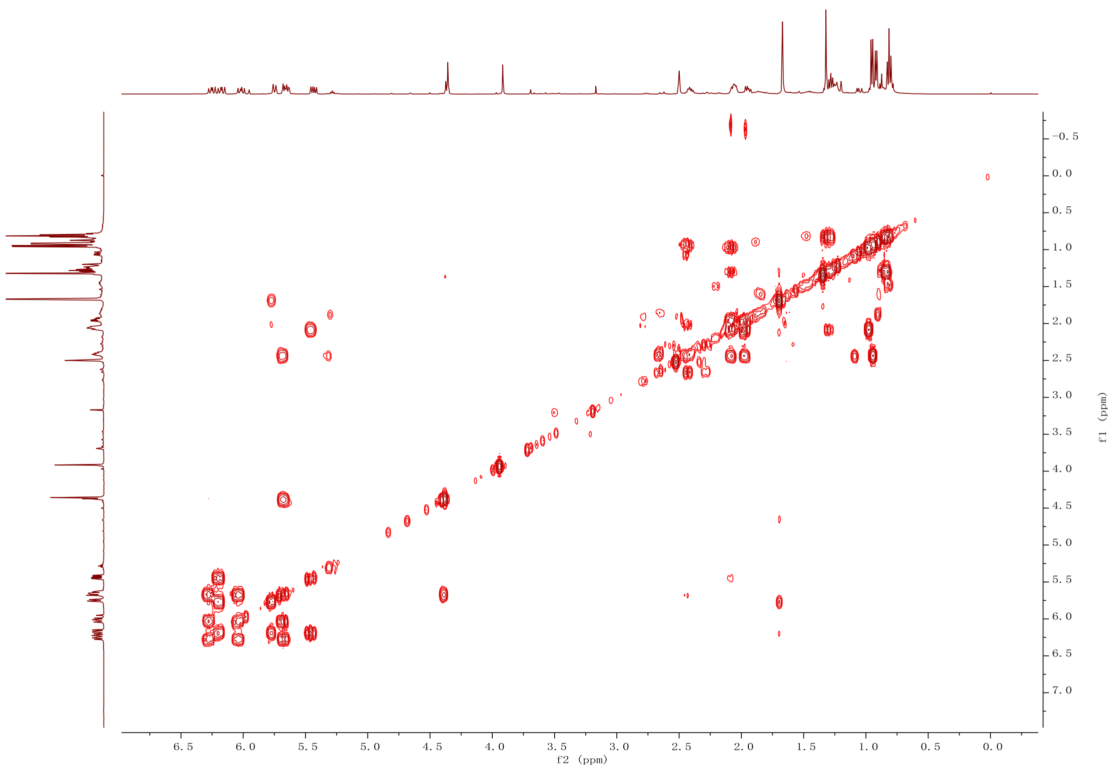

Figure S5. ^1H–^1H COSY spectrum of **1** in DMSO-d_6.

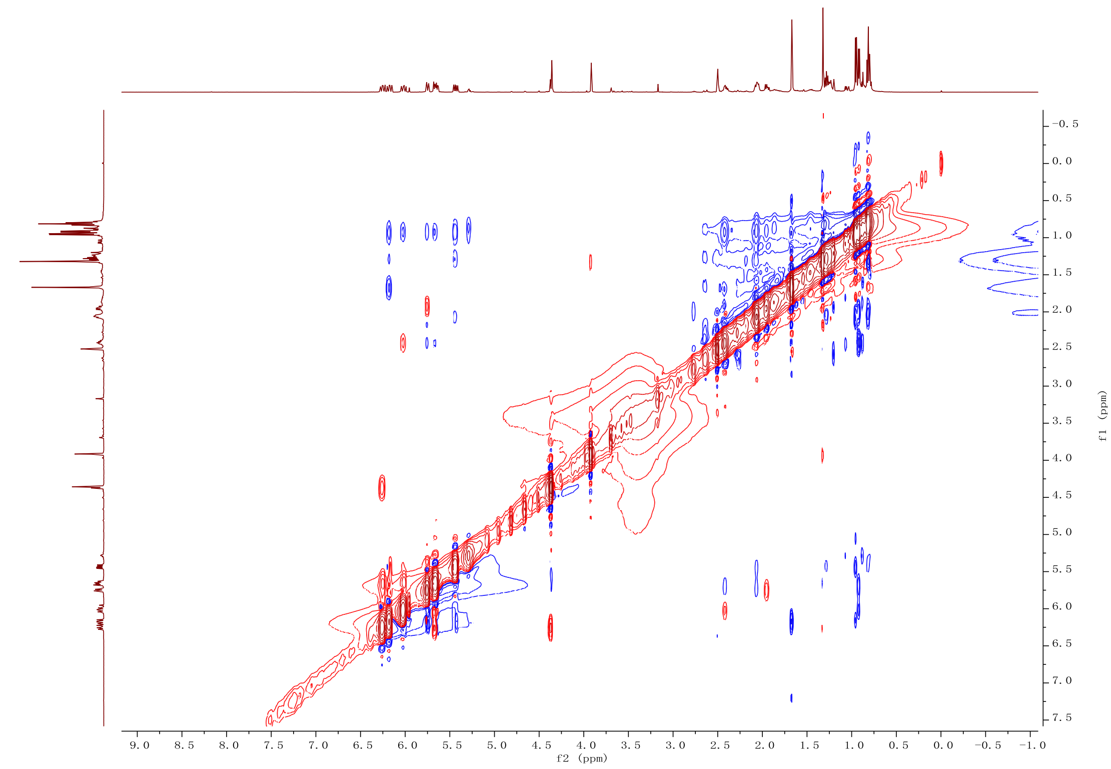

Figure S6. NOESY spectrum of **1** in DMSO-d_6.

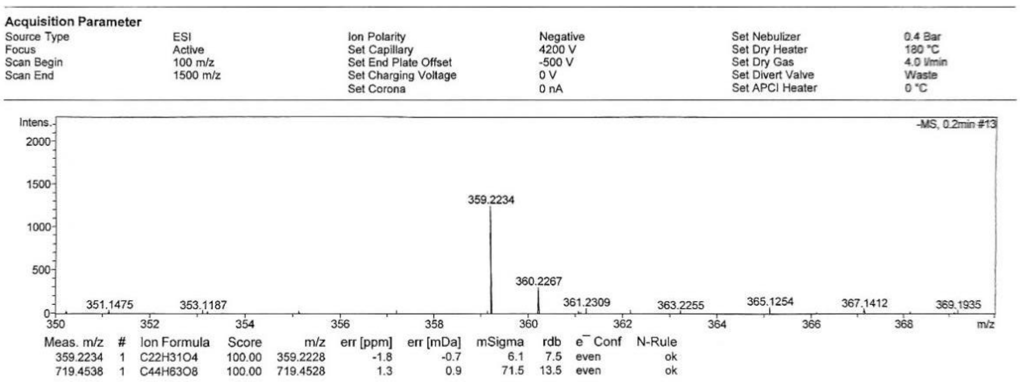

Figure S7. HRESIMS spectrum of **1**.

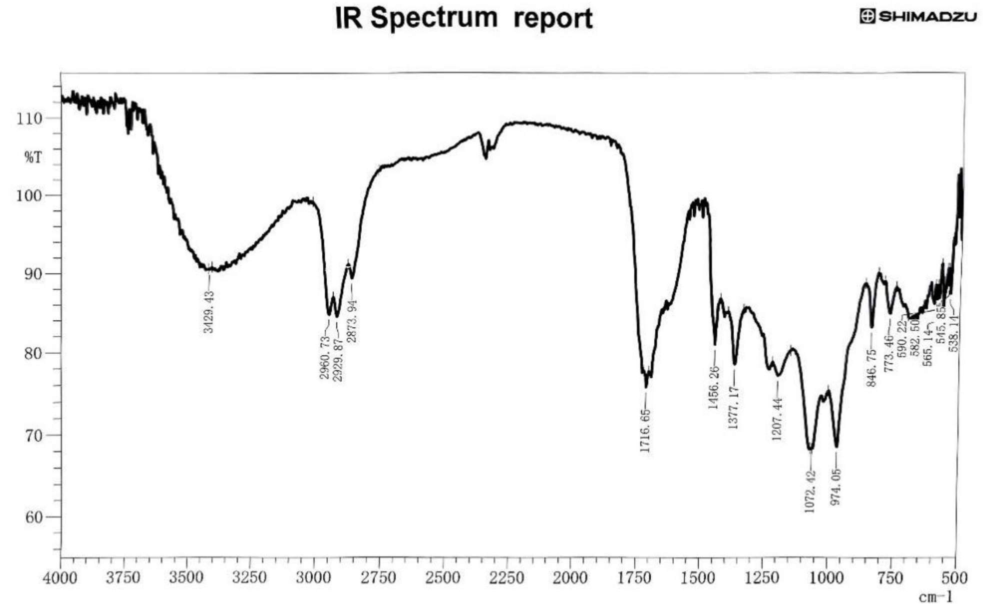

Figure S8. IR spectrum of **1**.

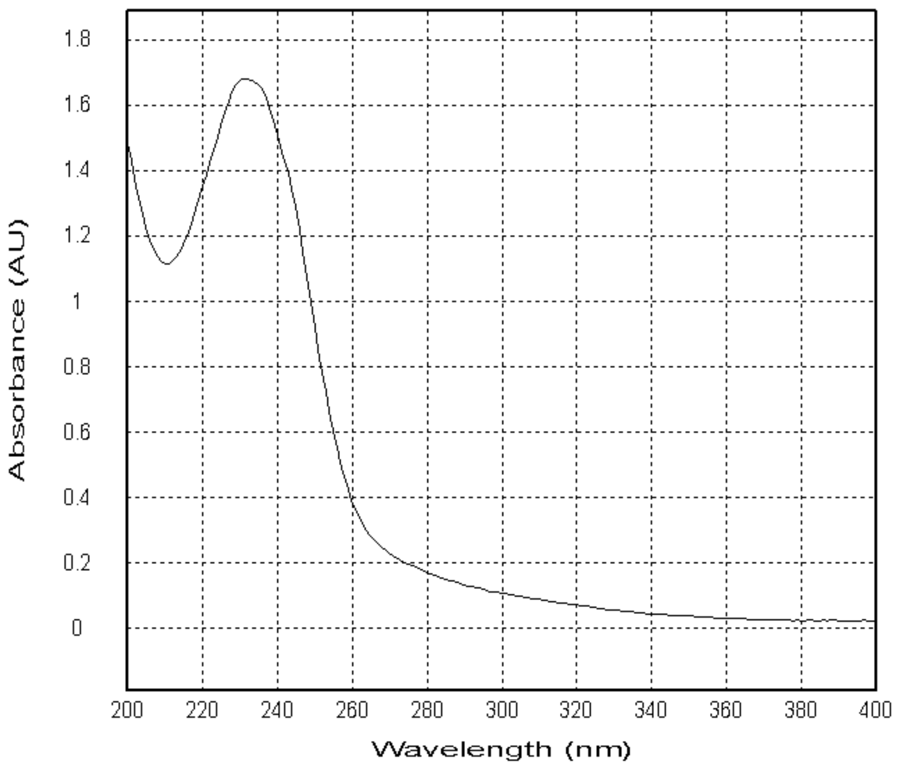

Figure S9. UV spectrum of **1** in MeOH.

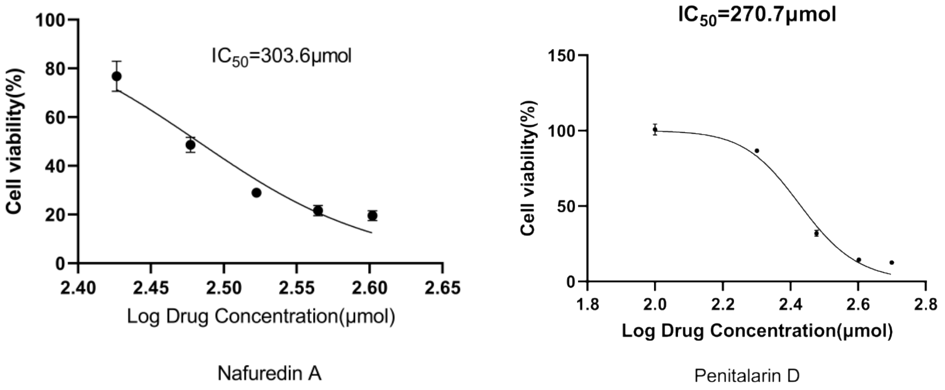

Figure S10. IC_{50} of penitalarin D (**1**) and nafuredin A (**3**) on HepG2 cells

Table S1. Primers of RT-qPCR

Primer	Sequence (5’→3’)
H-HMOX1-F	AAGACTGCGTTCCTGCTCAAC
H-HMOX1-R	AAAGCCCTACAGCAACTGTCG
H-Nrf2-F	TCAGCGACGGAAAGAGTATGA
H-Nrf2-R	CCACTGGTTTCTGACTGGATGT
H-FTH1-F	CCCCCATTTGTGTGACTTCAT
H-FTH1-R	GCCCGAGGCTTAGCTTTCATT
H-SLC7A11-F	TCTCCAAAGGAGGTTACCTGC
H-SLC7A11-R	AGACTCCCCTCAGTAAAGTGAC
H-GCLC-F	GGAGGAAACCAAGCGCCAT
H-GCLC-R	CTTGACGGCGTGGTAGATGT
H-GCLM-F	GTCTTGGAATGCACTGTATCTC
H-GCLM-R	CCCAGTAAGGCTGTAAATGCTC

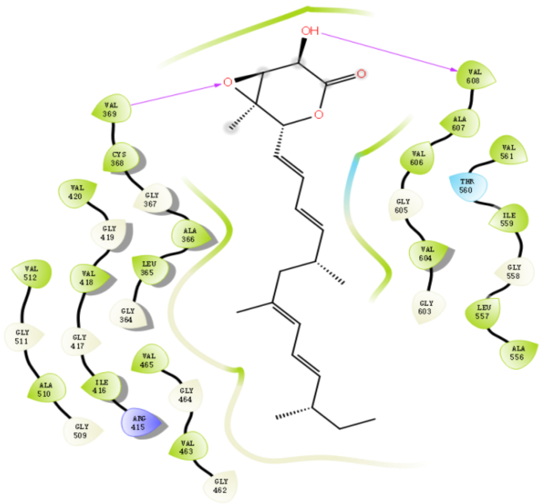

Figure S11. The docking site of the Nafuredin A complex with Nrf2-keap1

Table S2. The docking scores of the Nafuredin A complex with Nrf2-Keap1

Small-molecule ligands	Docking scores	PDB
nafuredin A	-5.352	5WHL
Hydroxyphenylpropionic acid	-6.012	5WHL
